# Unraveling the Context-Dependent Effects of Microplastics on Soil Enzymatic Activities via Interpretable Machine Learning

**DOI:** 10.3390/biology15201880

**Published:** 2026-10-18

**Authors:** Zifei Liu, Yitong Liu, Tianpeng Wang, Juanjuan Li, Xue Chi, Hong Li

**Affiliations:** Hainan Province Key Laboratory of One Health, Collaborative Innovation Center of Life and Health, School of Life and Health Sciences, Hainan University, Haikou 570228, China

**Keywords:** microplastics, soil enzymes, machine learning, model interpretability

## Abstract

**Simple Summary:**

Microplastics (MPs) are widespread in agricultural soils, but their effects on soil enzymatic activities vary considerably among studies. We used an interpretable machine learning framework to analyze a comprehensive dataset compiled from published studies and identify patterns associated with MP characteristics. Particle size showed the strongest model-based contribution, with a dataset-derived range of 200–600 μm associated with more pronounced predicted inhibition. Exposure time and dosage also showed non-linear patterns, including a plateau-like response at dosages above approximately 100 g/kg. Overall, our findings highlight the importance of considering MP size, exposure time, dosage, and polymer type when evaluating their potential effects on soil enzymatic activities and provide testable hypotheses for future experimental studies.

**Abstract:**

Microplastics (MPs) increasingly accumulate in agricultural soils, yet their effects on soil enzymatic activity remain heterogeneous. Here, a literature-derived dataset was analyzed using an interpretable machine learning framework. eXtreme Gradient Boosting (XGBoost) and Random Forest (RF) captured non-linear patterns in enzymatic responses to MP characteristics. Particle size showed the strongest model-based contribution, with a dataset-derived size-sensitive range of 200–600 μm associated with more pronounced predicted inhibition. Dosage showed a plateau-like pattern above approximately 100 g/kg, while exposure time exhibited non-linear variation across the observed duration. SHapley Additive exPlanations (SHAP) analysis further characterized the relative contributions of individual features to model predictions. Overall, the results provide quantitative, data-driven insights into heterogeneous MP-soil enzyme associations and highlight particle size as an important factor for further experimental investigation and risk assessment.

## 1. Introduction

Microplastics (MPs), plastic particles smaller than 5 mm, have become pervasive contaminants across global ecosystems, originating from both primary industrial materials and the fragmentation of larger plastic debris. They have been detected in diverse environmental compartments, from the atmosphere to deep-sea sediments [1,2]. In recent years, agricultural soils have been increasingly recognized as a major sink for MP accumulation. Intensive agricultural practices, such as plastic mulching, wastewater irrigation, and sewage sludge application, introduce substantial quantities of MPs into soil systems, often resulting in concentrations that exceed those reported in marine environments [3,4]. Consequently, understanding how MPs influence soil ecological processes has become a central issue in environmental research.

Soil extracellular enzymes play a fundamental role in mediating nutrient cycling and organic matter decomposition, and are widely used as sensitive indicators of soil functional status. Numerous studies have investigated the effects of MP exposure on enzymatic activities; however, the reported responses vary considerably across experimental conditions [5,6,7]. Existing studies generally report three types of outcomes: (1) inhibitory effects, often associated with microbial stress or physicochemical interference; (2) stimulatory effects, potentially linked to shifts in microbial activity or resource availability; and (3) mixed or condition-dependent responses. This variability is largely driven by the combined influence of MP characteristics, including polymer type, particle size, dosage, and exposure time, which shape enzymatic responses under different conditions. Importantly, this inconsistency limits our ability to predict how MP contamination influences soil ecosystem functioning and complicates ecological risk assessment across environmental scenarios. Although conventional meta-analyses have identified general trends, they are often insufficient to capture complex relationships among multiple interacting variables [5,6]. As a result, a quantitative understanding of how MP characteristics collectively determine both the direction and magnitude of enzymatic responses across environmental conditions remains limited.

To address these challenges, machine learning (ML) provides a powerful framework for capturing non-linear interactions among multiple exposure factors in heterogeneous datasets. Algorithms such as Random Forest (RF) and eXtreme Gradient Boosting (XGBoost) are particularly suitable for identifying interaction effects and threshold behaviors [8]. In environmental and soil science, ML has been increasingly applied to identify threshold responses of microbial activity to heavy metals, to predict enzyme inhibition under mixed pollutant exposure, and to quantify the relative contributions of multiple stressors to soil biological processes [9]. Such capabilities are particularly relevant for MP-soil systems, where multiple interacting factors jointly influence biological responses. Importantly, the integration of ML with interpretable analytical tools enables not only accurate prediction but also insight into underlying response mechanisms [10]. Feature importance analysis allows the identification of dominant predictors, while Partial Dependence Plots (PDPs) reveal non-linear response patterns and potential threshold ranges [11]. In addition, SHapley Additive exPlanations (SHAP) provide a quantitative decomposition of model outputs at the sample level, enabling the assessment of how individual variables contribute under specific conditions [12]. Together, these approaches facilitate the interpretation of context-dependent interactions among MP properties.

In this study, we developed an interpretable ML framework to systematically evaluate the effects of MPs on soil enzymatic activity using a comprehensive dataset compiled from published studies. MP characteristics, including polymer type, particle size, dosage, and exposure time, into non-linear models; we aimed to resolve complex response patterns that cannot be captured by conventional linear approaches. Specifically, using a literature-derived observational dataset, our objectives were to (1) quantify the relative importance of different MP properties in determining both the direction and magnitude of enzymatic responses; (2) identify non-linear response patterns and potential threshold ranges associated with key variables; and (3) elucidate how combinations of MP characteristics jointly influence enzymatic activity under different conditions. This work provides a quantitative and interpretable framework for understanding MP-soil interactions and offers insights for ecological risk assessment and environmental management in MP-contaminated agricultural systems.

## 2. Materials and Methods

### 2.1. Data Collection

To investigate the impact of MP properties on soil biological health, a systematic literature search was conducted in the Web of Science database in two rounds: an initial search in May 2023 (covering publications from 2012 to 2022) and an updated search in May 2025 (covering publications from 2023 to 2024) (Figure S1). Both searches used the following Boolean query: TS = (“microplastic*” OR “MP*”) AND (“soil propert*” OR “soil physical propert*” OR “soil chemical propert*” OR “soil physicochemical propert*”) AND (“soil enzyme*” OR “enzyme activit*” OR “soil enzymatic activit*”). Both searches were limited to peer-reviewed journal articles published in English. Duplicate records were removed using EndNote X9. Screening was performed by one reviewer, with uncertain cases discussed with a second reviewer.

The initial pool of articles was refined based on the following inclusion criteria:

(1) explicit specification of MP characteristics, including type (restricted to PP, PE, PS, PLA, or PVC; Table S1), size, dose, and exposure time;
(2) comprehensive reporting of soil enzyme categories and their respective activities;
(3) documentation of soil origins to ensure data traceability, although these data were not used as analytical predictors.

Studies failing to meet any of these criteria were excluded.

Given that the vast majority of studies meeting these stringent criteria originated from China, the final dataset was restricted to studies conducted within this region. Consequently, 19 peer-reviewed studies were obtained, yielding a curated dataset of 592 data entries (Table S2). The dataset encompasses 17 key enzymes (along with one general phosphatase entry lacking a specific subtype) involved in Carbon (C), Nitrogen (N), and Phosphorus (P) cycling, such as acid phosphatase, alkaline phosphatase, neutral phosphatase, phosphatase, β-glucosidase, FDAse, urease, N-acetyl-β-glucosaminidase, β-xylosidase, cellulase, leucine aminopeptidase, phenol oxidase, catalase, invertase, polyphenol oxidase, sucrase, and laccase. For data presented solely in graphical format, numerical values were extracted using GetData Graph Digitizer (version 2.2), and a random subset was cross-checked against the original figures.

### 2.2. Data Preprocessing

#### 2.2.1. Feature Standardization

Given the substantial differences in the numerical scales of MP size, dose, and exposure time, Z-score standardization was applied to these continuous features. This process ensures that each feature contributes equally to the model training. The standardization was calculated as follows:

(1) $$Z=\frac{X-\mu }{\sigma }$$

where Z represents the standardized feature value, X is the raw feature value in its original scale (MP size in μm, dose in g/kg, and exposure time in days), and μ and σ denote the mean and standard deviation of the feature, respectively. This procedure was implemented using the scikit-learn library (version 1.3.2) in Python 3.13.5 [13], an open-source toolkit commonly utilized for data preprocessing and feature scaling.

#### 2.2.2. Enzyme Activity Calculation

To normalize baseline variations across different soil enzymes, the relative enzyme activity (E) was defined as follows:

(2) $$E=\frac{x_{t}}{x_{c}}$$

where x_t_ and x_c_ represent the enzyme activities in the MP-treated and control groups, respectively. In this study, E values ranged from 0 to 2. To facilitate qualitative analysis, the response was further dichotomized into a classification target: E > 1 was defined as promotion, while E ≤ 1 was categorized as inhibition.

#### 2.2.3. Data Splitting

The dataset was randomly divided into the training set (90%) and independent test set (10%). All model training and hyperparameter optimization were performed using the training set, while the independent test set was reserved exclusively for final performance evaluation.

### 2.3. Model Training and Optimization

To comprehensively evaluate the impacts of microplastics on soil enzymatic activity, we established a dual-path ML framework comprising regression and classification tasks. Five representative ML algorithms were employed for regression (quantitative) analysis, including Support Vector Regression (SVR), Gradient Boosting Regression (GBR), RF, Adaptive Boosting (AdaBoost), and XGBoost. For qualitative classification, an RF-based classifier was implemented to distinguish between enzymatic promotion and suppression. A total of four MP properties as input features. Specifically, size, dosage, and exposure time were treated as continuous numerical values, while type was processed using label encoding. The regression model predicted continuous enzymatic activity values, whereas the classification model predicted binary labels (promotion vs. inhibition).

Both regression and classification models were trained using five-fold cross-validation, with hyperparameter optimization performed using RandomizedSearchCV in the scikit-learn library (version 1.3.2). RandomizedSearchCV randomly samples a specified number of parameter combinations from predefined search spaces, evaluates each combination using cross-validation, and selects the best-performing combination as the final configuration.

The hyperparameter search spaces for each algorithm were defined as follows:

XGBoost: max_depth [3, 4, 5, 6, 7, 8, 9, 10], learning_rate [0.01, 0.05, 0.1, 0.15, 0.2], n_estimators [50, 100, 200, 500, 1000], min_child_weight [1, 2, 3, 4], gamma [0, 0.1, 0.2, 0.3, 0.4], and both subsample and colsample_bytree [0.5, 0.6, 0.7, 0.8, 0.9].

AdaBoost: n_estimators [50, 100, 150, 200] and learning_rate [0.1, 0.2, 0.3].

SVR: RBF kernel, with regularization parameter C [0.1, 1, 10, 100], γ [0.01, 0.1, 1, 10], and ε [0.01, 0.1, 1].

GBR: n_estimators [50, 100, 200, 500, 1000], learning_rate [0.01, 0.05, 0.1, 0.15, 0.2], max_depth [3, 4, 5, 6, 7, 8, 9, 10], min_samples_split [2, 20], min_samples_leaf [1, 20], subsample [0.5, 0.6, 0.7, 0.8, 0.9].

RF (including both regression and classification models): n_estimators [50, 100, 200, 500, 1000], max_features [‘sqrt’, ‘log2’], max_depth: [3, 4, 5, 6, 7, 8, 9, 10], min_samples_split [2, 20], min_samples_leaf [1, 20].

All algorithms were implemented using the scikit-learn library (version 1.3.2).

In addition, two MATLAB-based neural-network benchmarks were used under five-fold cross-validation. Both the neural net fitting tool (NFT, MATLAB R2024a) for regression and the neural pattern recognition tool (NPRT, MATLAB R2024a) for classification shared the same hyperparameter search space: hidden layer sizes {5, 10, 15, 20, 25, 30, [10 5], [15 10]} and training algorithms {‘trainscg’, ‘trainrp’}. Both models used a fixed random seed of 42 (rng(42)) to ensure reproducibility.

### 2.4. Model Evaluation

The performance of regression models was evaluated using two complementary metrics: Root Mean Square Error (RMSE) and Mean Absolute Error (MAE), implemented using the mean_squared_error and mean_absolute_error functions in the scikit-learn library (version 1.3.2). RMSE squares prediction errors before averaging, placing greater weight on large deviations. This is particularly relevant for multi-source environmental datasets with high between-study variability, where minimizing large prediction errors is critical. MAE, by contrast, offers a robust view of typical prediction accuracy by measuring the average absolute difference with less sensitivity to outliers. Lower RMSE and MAE values indicate better predictive accuracy. Both metrics were considered jointly; their consistent model rankings further reinforce the reliability of the selected models. The two metrics were calculated as follows:

(3) $$\mathrm{RMSE}=\sqrt{\frac{1}{m}\sum_{i=1}^{m}{\left(y_{i}-t_{i}\right)}^{2}}$$

(4) $$\mathrm{MAE}=\frac{1}{m}\sum_{i=1}^{m}\left|y_{i}-t_{i}\right|$$

where *t_i_* and *y_i_* denote the actual and predicted soil enzyme activities for the *i*-th sample, respectively, and m is the total number of samples.

For the classification task, model performance was assessed using the Area Under the Receiver Operating Characteristic Curve (AUC-ROC), which reflects the model’s ability to distinguish between enzymatic promotion and inhibition. Higher AUC values indicate better classification performance [14].

### 2.5. Interpretability Analysis

To reveal how each MP property contributes to enzymatic responses, three complementary interpretability approaches were employed. Global feature importance was derived from the RF classifier, calculated as the mean decrease in impurity (MDI) averaged across all trees [15]. This ranking identified the contribution of each MP property to the directional shift (promotion vs. inhibition) of enzymatic activity. PDPs were generated for each continuous feature (MP size, exposure time, and dosage) using three algorithms (XGBoost, RF, and GBR) to visualize the marginal effect of each feature on the predicted enzymatic activity [10]. The consistency of PDP trends across different algorithms was examined to ensure consistency across the three models. SHAP analysis was applied to the XGBoost regression model [12]. SHAP values quantify the contribution of each feature to individual predictions. Summary plots provide global interpretability by ranking feature importance, while force plots illustrate the factors driving specific predictions for individual samples. It should be noted that the RF-based MDI and the XGBoost-based SHAP framework serve complementary roles within this analytical design. Specifically, MDI reflects tree-splitting frequency and structural impurity reduction to provide a qualitative stability check, whereas SHAP values quantify game-theoretic marginal contributions to serve as our primary metric for continuous regression outputs. Together, these two approaches provide a multi-faceted evaluation of feature importance while accounting for their inherent algorithmic differences.

## 3. Results

### 3.1. Weak Linear Correlations Underscore the Complexity of MP-Soil Enzyme Interactions

**Figure 1:**
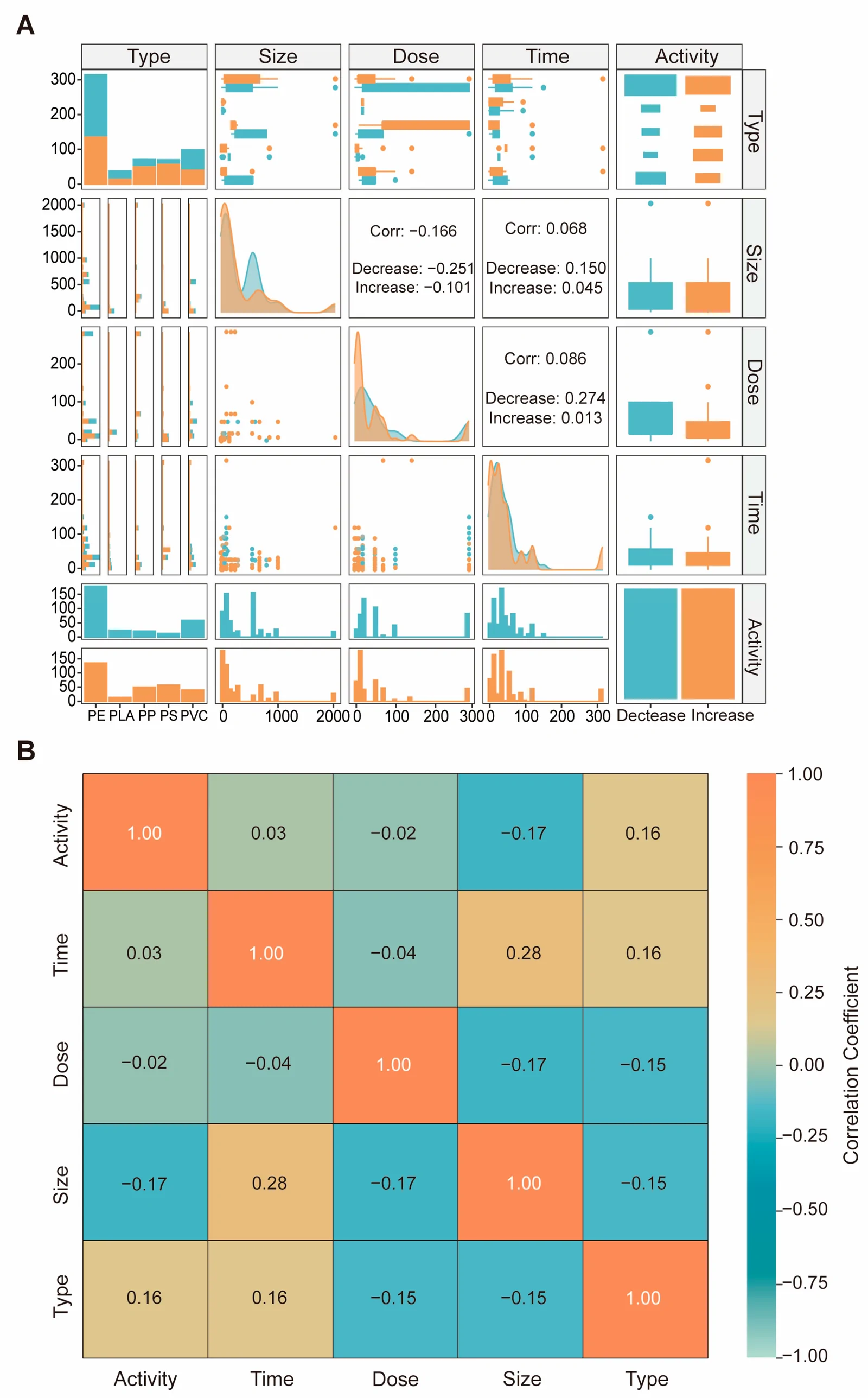
Multivariate statistical analysis of microplastic characteristics and soil enzymatic activity. (**A**) Pairwise distribution and correlation matrix of the dataset (*n* = 592). The diagonal panels display the density distribution of each variable, including microplastic (MP) type, size, dosage, exposure time, and enzymatic activity. Color coding indicates the directional response: blue for inhibition (Decrease) and orange for promotion (Increase). (**B**) Heatmap of Spearman correlation coefficients among MP characteristics and soil enzymatic activity. The color scale represents the strength of correlation, ranging from −1.00 (blue, negative) to 1.00 (orange, positive).

Initial exploration of the compiled dataset (Figure 1A) demonstrated a wide and heterogeneous distribution of MP properties, where experimental data points for enzymatic promotion and inhibition were highly overlapped across the entire ranges of MP size, dose, and exposure time. Specifically, density plots and histograms revealed a heavy-tailed distribution for MP size, with most data concentrated below 1000 μm; however, enzymatic responses varied drastically even within this narrow window. Similarly, both inhibitory and promotional effects were observed across a broad spectrum of dose (0–280 g/kg) and exposure time (0–310 days), yet no distinct clusters or linear separating boundaries were visible in the pairwise scatter plots. Furthermore, while PE accounted for the largest proportion of data entries, all five polymer types—PE, PLA, PP, PS, and PVC—exhibited a mixture of both increased and decreased enzymatic activities, suggesting that individual MP properties do not exert a simple, monotonic influence on soil biological health.

This visual complexity was further confirmed by Pearson correlation analysis (Figure 1B). The low inter-variable correlation coefficients (typically |r| < 0.2) suggested weak linear associations among the four MP properties. Furthermore, a striking lack of linear correlation was observed between enzyme activity and any individual MP property, with correlation coefficients for MP type, size, dose, and exposure time being 0.16, −0.17, −0.02, and 0.03, respectively (Figure 1B). These near-zero values indicated that the biological response of soil to MP stress was not governed by simple linear pathways. This highlighted the inherent non-linear nature of MP interference, providing a strong scientific justification for employing advanced ML architectures.

### 3.2. XGBoost and RF Achieve High Performance in Enzymatic Prediction and Classification

**Figure 2:**
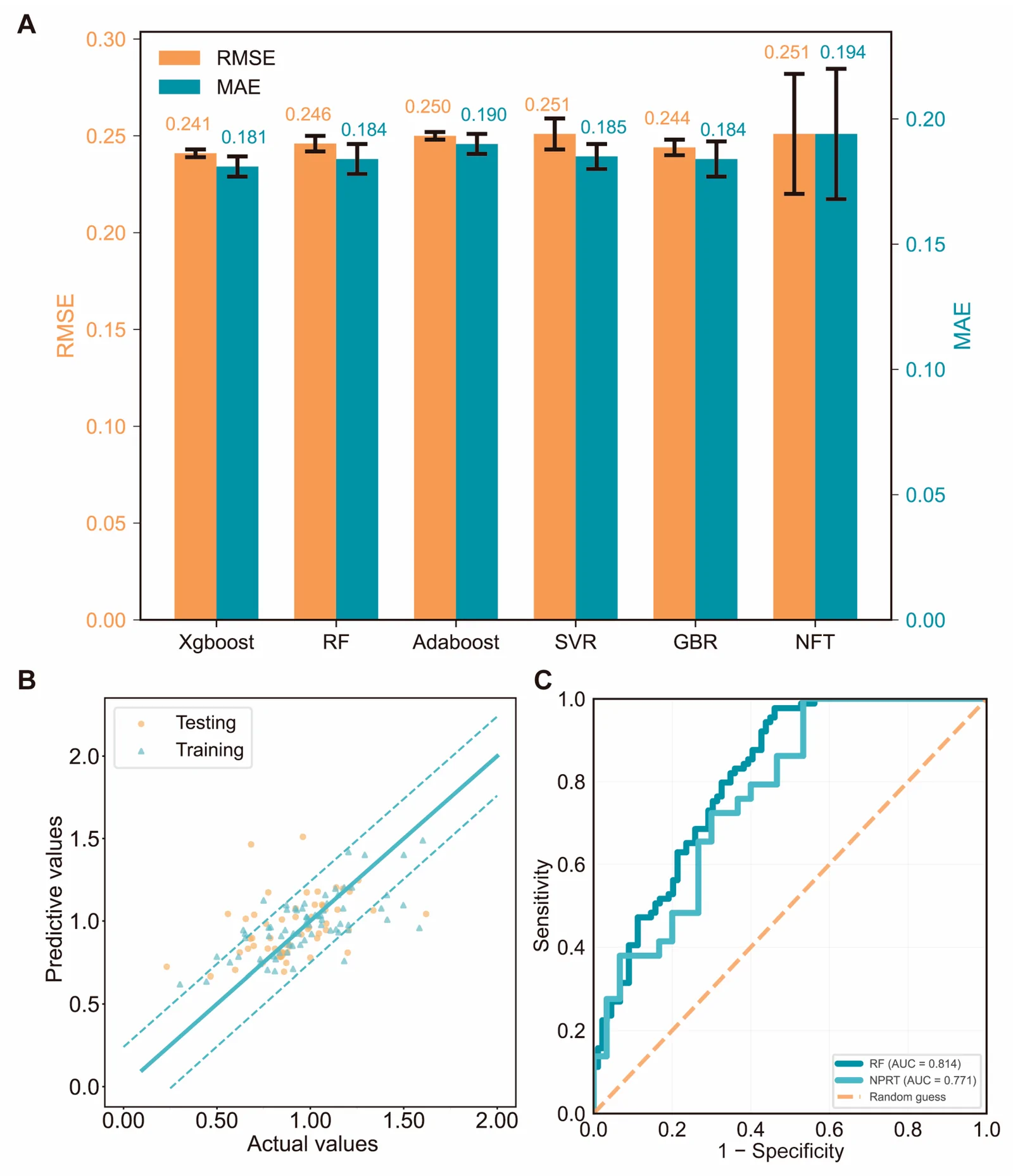
Predictive performance of machine learning models. (**A**) Comparison of regression algorithms. The bar chart displays the Root Mean Square Error (RMSE) and Mean Absolute Error (MAE) for six different models (XGBoost, RF, AdaBoost, SVR, GBR, and NFT). Error bars represent the standard deviation derived from five-fold cross-validation. (**B**) Fitting proficiency of the XGBoost model. The solid line represents the 1:1 ideal fit, while dashed lines represent ±1 × RMSE (0.241) around the 1:1 line, serving as a visual reference for the typical prediction error magnitude. (**C**) Receiver Operating Characteristic (ROC) curve. The classification performance is quantified by the Area Under the Curve for distinguishing the directional responses (inhibition vs. promotion) of soil enzymatic activity.

To test whether non-linear ML models could capture the complex MP-enzyme relationships, the four MP properties were utilized as input features to predict the continuous response of soil enzymatic activity. Six models were evaluated: five scikit-learn regression algorithms (SVR, GBR, RF, AdaBoost, and XGBoost) and one MATLAB-based neural-network benchmark (NFT). Among the six models, XGBoost exhibited the highest predictive precision, achieving the lowest error metrics (RMSE: 0.241; MAE: 0.181) (Figure 2A) and the highest coefficient of determination (R^2^) (0.293 ± 0.042) (Table S3). To rigorously evaluate the robustness of XGBoost and ensure that these outcomes were not artifacts of a single favorable random seed, a comprehensive test using *n* = 50 independent random seeds was performed. The results yielded a stable performance distribution with a mean RMSE of 0.271 (ranging from 0.213 to 0.319) and a mean MAE of 0.202 (ranging from 0.162 to 0.236). The reliability of the XGBoost model was further validated by the regression scatter plot (Figure 2B). The results showed that the predicted values aligned closely with actual observations, with both training and testing data clustered tightly along the 1:1 diagonal line, showing no signs of overfitting despite the diversity of data sources.

Beyond numerical regression, we further conducted a qualitative assessment. An RF classifier was developed to distinguish between enzymatic promotion and inhibition, and an NPRT was evaluated as a neural-network benchmark on the same independent test set. The RF classifier achieved an AUC of 0.814, while the NPRT achieved an AUC of 0.771 (Figure 2C), indicating that the RF model provided stronger discriminative performance in the present dataset. To further evaluate the stability of the classification performance, the RF model was evaluated across 50 independent random seeds, yielding a mean AUC of 0.819 (ranging from 0.740 to 0.858), indicating robustness to random initialization. Together, these results indicate that the ensemble-based RF approach can effectively capture categorical response patterns, complementing the numerical predictions obtained from XGBoost.

An in-sample one-way variance decomposition revealed that source DOI accounted for the largest share of data variance (η^2^ ≈ 0.391), compared with size (η^2^ ≈ 0.348), time (η^2^ ≈ 0.212), dose (η^2^ ≈ 0.190), and type (η^2^ ≈ 0.096). Although these were non-causal estimates, they underscored that source identity was a dominant structural factor. To ensure strict scientific independence, we supplemented our evaluation using a 19-fold Leave-One-Group-Out Cross-Validation (LOGO-CV) grouped by DOI. Under this scheme, all records originating from the same literature source were strictly isolated within the same fold. As expected, the LOGO-CV yielded lower performance metrics due to the strict separation of literature sources. Across the 19 folds, the XGBoost regression model achieved a mean RMSE of 0.318 ± 0.108 and a mean MAE of 0.269 ± 0.098 (Table S4). Study-level bootstrap resampling yielded 95% confidence intervals of [0.271, 0.371] for RMSE and [0.225, 0.317] for MAE. The RF classification model yielded an AUC of 0.530.

### 3.3. Feature Importance Reveals MP Size as the Dominant Driver of Enzymatic Shifts

**Figure 3:**
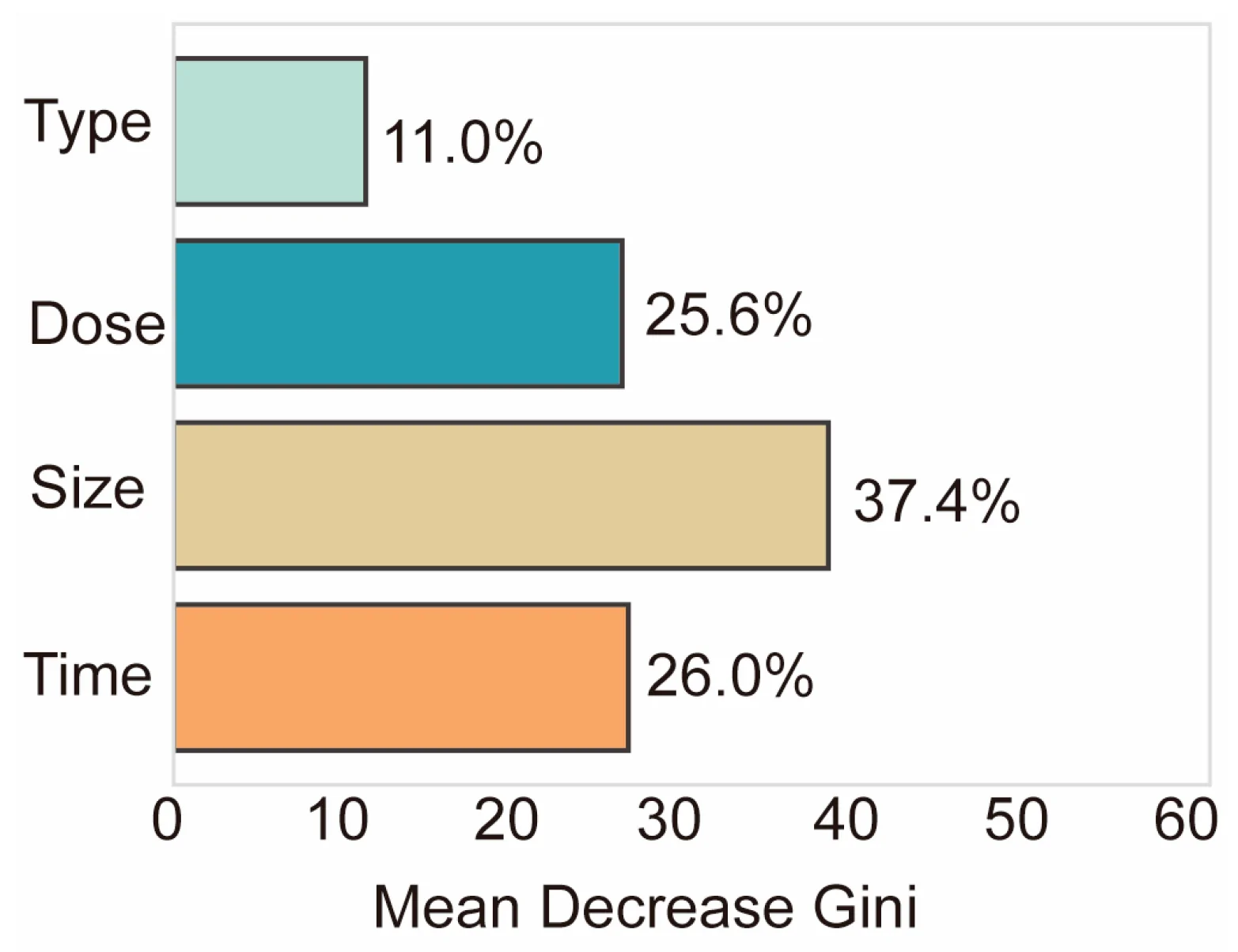
Feature importance analysis. Relative importance of microplastic (MP) characteristics. Feature importance is quantified by the Mean Decrease Gini (MDI) using the Random Forest model, with higher values indicating greater influence on model predictions.

To identify the key drivers of enzymatic shifts, we next performed global feature importance analysis based on the RF classifier trained on the random-split dataset. The importance ranking identified MP size (37.4%) as a leading feature associated with the directional shift in enzymatic activity, followed by exposure time (26.0%) and dosage (25.6%) (Figure 3). In contrast, MP type (11.0%) contributed the least to the model’s overall discriminative power. To rigorously address potential literature-level heterogeneity and MDI metric bias, we further implemented a LOGO-CV permutation importance analysis within the 19-fold LOGO-CV (Table S5). Consistent with the MDI results, size emerged as a primary robust predictor, confirming that the dominant role of size is stable across different data splitting strategies and importance quantification methods (Figure S2).

### 3.4. PDP Reveals Non-Linear Thresholds for Size, Exposure Time, and Dosage

**Figure 4:**
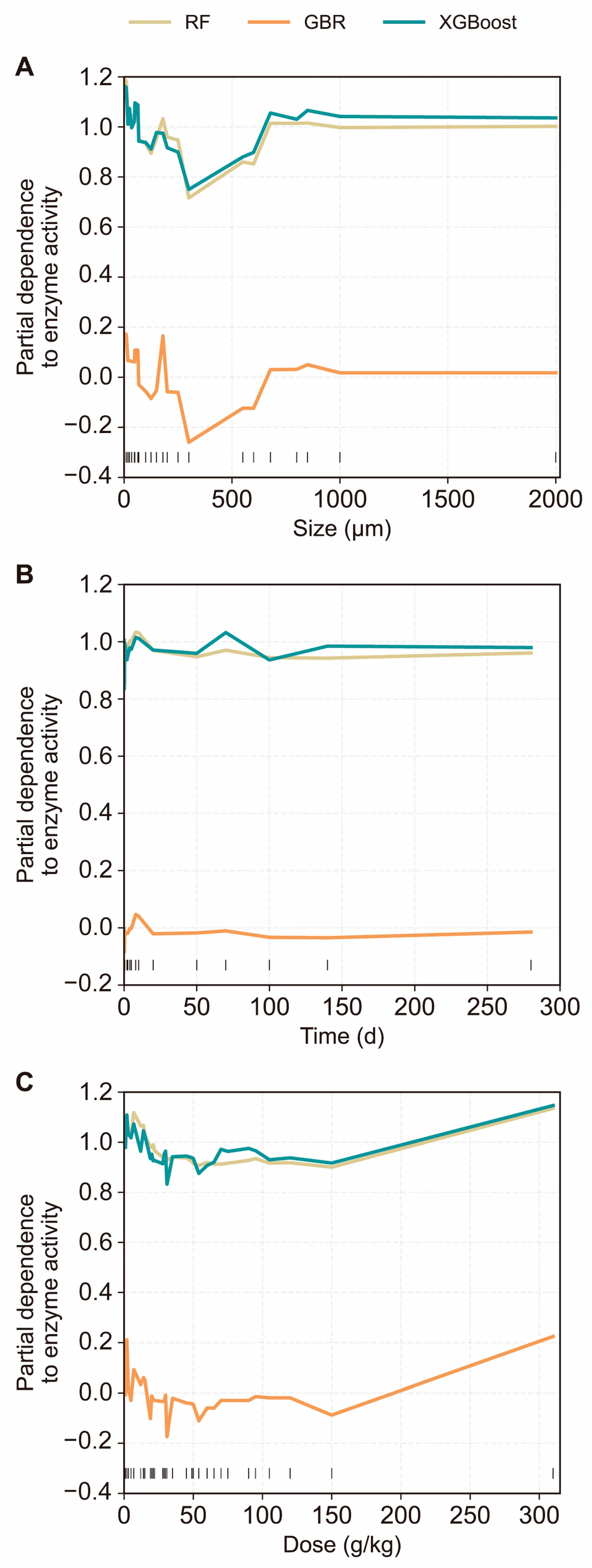
Partial dependence plots (PDP) of microplastic characteristics on soil enzymatic activity. The panels illustrate the marginal effects of (**A**) particle size, (**B**) exposure time, and (**C**) dosage on the predicted enzymatic response. The lines represent the partial dependence calculated from three machine learning models: Random Forest (RF), Gradient Boosting Regressor (GBR), and eXtreme Gradient Boosting (XGBoost). The *y*-axis denotes the partial dependence to enzyme activity, reflecting the change in the average prediction as a specific input variable varies while other features are marginalized. Vertical lines at the bottom indicate the actual data support.

To further dissect how each MP property influences enzymatic activity, we generated PDPs for size, exposure time, and dosage using three distinct algorithms: XGBoost, RF, and GBR trained on the random-split dataset (Figure 4 and Tables S6–S8). The relationship between MP size and enzymatic activity (Figure 4A) emerged as the most critical and complex, revealing a “size-sensitive window” for MP toxicity. The strongest inhibitory effect (lowest PDP values) was consistently observed within the 200–600 μm range across all models (Figure 4A), supported by well-separated 95% confidence bands from Bootstrap resampling (1000 iterations) (Figure S3). This window was statistically validated using Kruskal–Wallis tests followed by Mann–Whitney U pairwise comparisons (all Benjamini–Hochberg-corrected *p* < 0.05; Table S9).

**Figure 5:**
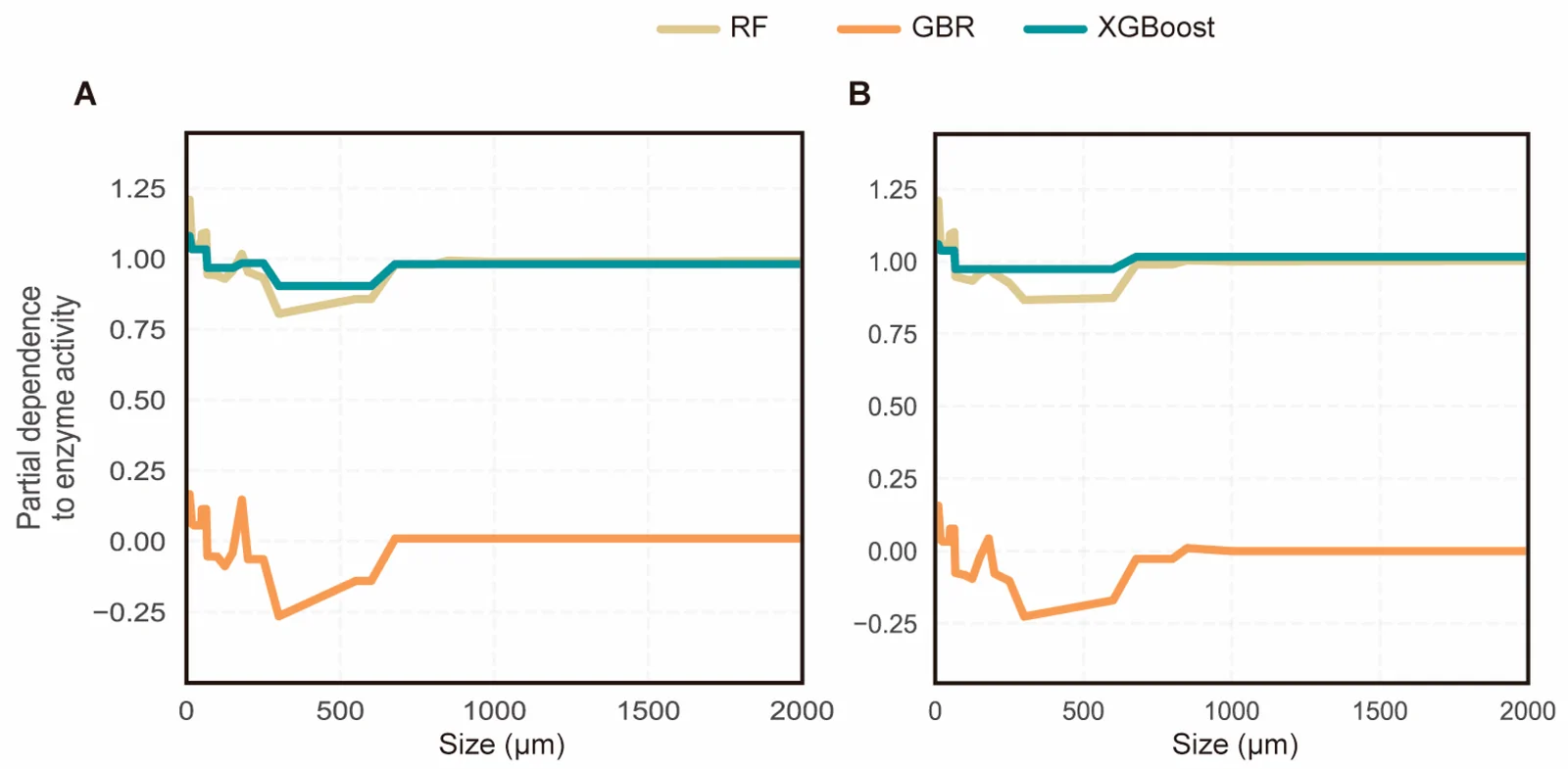
Robustness of the size partial dependence plots (PDP) under source-grouped validation. (**A**) PDP based on 19-fold Leave-One-Group-Out Cross-Validation (LOGO-CV) grouped by DOI. (**B**) PDP after excluding the dominant source (DOI 10.1016/j.chemosphere.2022.135836) and retraining on the remaining 18 studies. Curves represent RF, GBR, and XGBoost.

To assess whether this “size-sensitive window” was robust to source composition, we performed two additional analyses. First, we repeated the PDP analysis within a 19-fold LOGO-CV framework grouped by DOI. The PDP curves were nearly identical to those obtained from the random-split evaluation, with the 200–600 μm window clearly present (Figure 5A). Second, because the dominant source (DOI 10.1016/j.chemosphere.2022.135836) contributed 90 of 138 records within this window, we additionally excluded this study and repeated the analysis on the remaining 18 studies within the same framework. The 200–600 μm window remained present, although the PDP trough became shallower compared with the random-split result (Figure 5B). Together, these results indicate that the existence of the size-sensitive window is not driven by any single source, although its depth may be partially influenced by the dominant source.

Following size, exposure time (Figure 4B) also exhibited a distinct non-monotonic trend. Soil enzyme activity showed greater variability during the early exposure period (0–105 days), followed by a gradual upward trend toward 310 days. Because the compiled dataset was not longitudinal, these patterns should be interpreted as cross-study model predictions rather than temporal trajectories within individual experiments. Similarly, MP dosage (Figure 4C) showed a non-linear pattern, with greater variability at low dosages (≤20 g/kg), whereas changes in the predicted response were weaker at dosages above approximately 100 g/kg, indicating a plateau-like pattern in the model predictions. Similar results were also observed under the LOGO-CV framework (Figure S4). To further examine feature effects, we supplemented the PDP analysis with Accumulated Local Effects (ALE) plots (Figure S5). The ALE curves showed non-linear patterns consistent with the PDP results. Rug plots indicated that data support was sparse in high-value regions, and these regions should be interpreted with caution.

### 3.5. SHAP Uncovers Hierarchical Regulation and Context-Dependent Synergy

**Figure 6:**
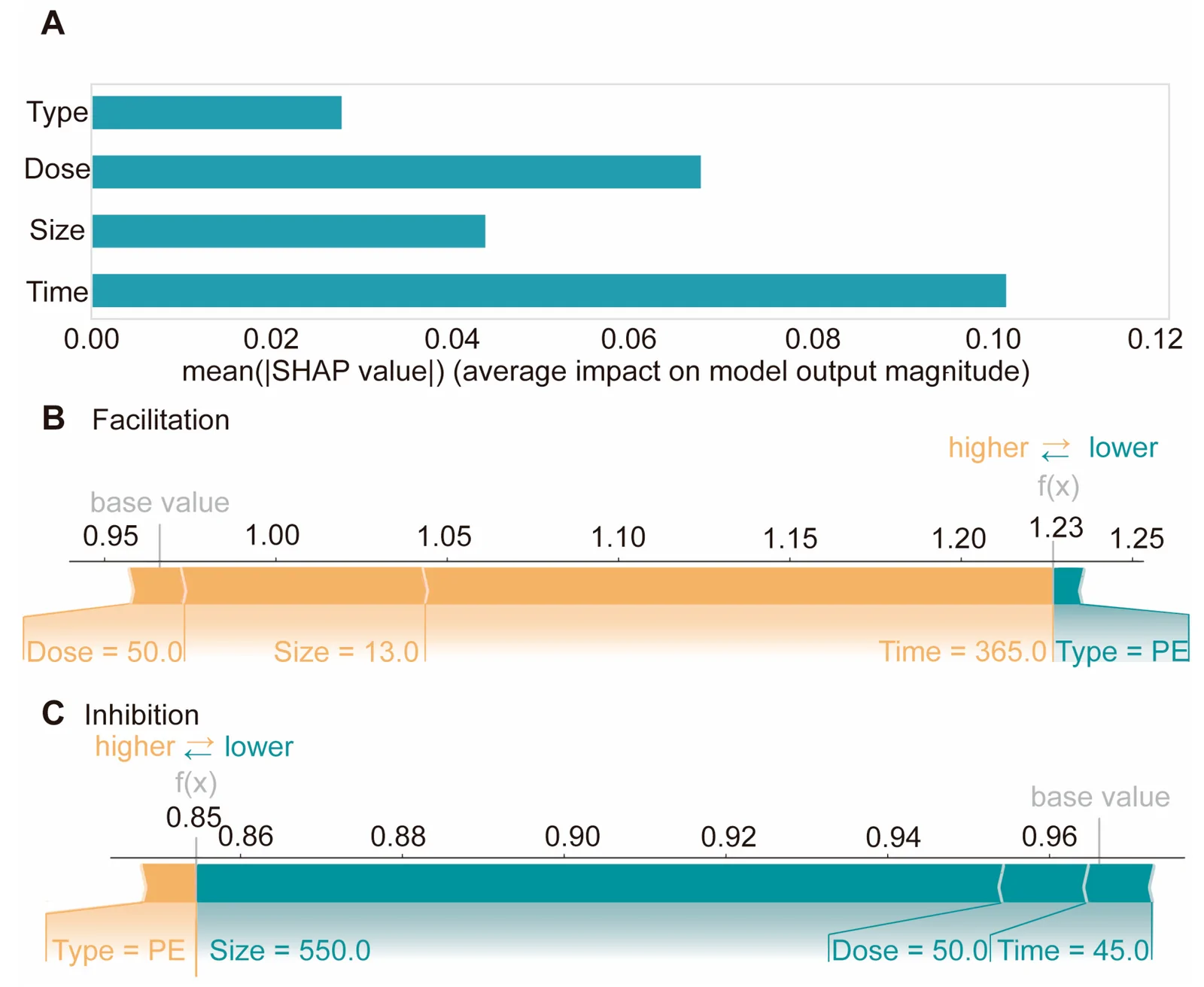
SHapley Additive exPlanations (SHAP) analysis of feature contributions. (**A**) Global feature importance based on the XGBoost model. The bar chart ranks features based on their mean absolute SHAP values, quantifying their average impact on the model’s prediction of enzymatic activity. (**B**) Force plot for a typical facilitation case. For an individual sample exhibiting enhanced enzymatic activity (base value = 0.97 and f(x) = 1.23), features such as dose, particle size, and exposure time (red arrows) contribute positively to the base value, while type (blue arrow) exerts a minor buffering effect. (**C**) Force plot for a typical inhibition case. For a sample showing suppressed activity (base value = 0.97 and f(x) = 0.85), specific parameters drive the prediction significantly below the base value, indicating their role in the inhibitory response.

**Figure 7:**
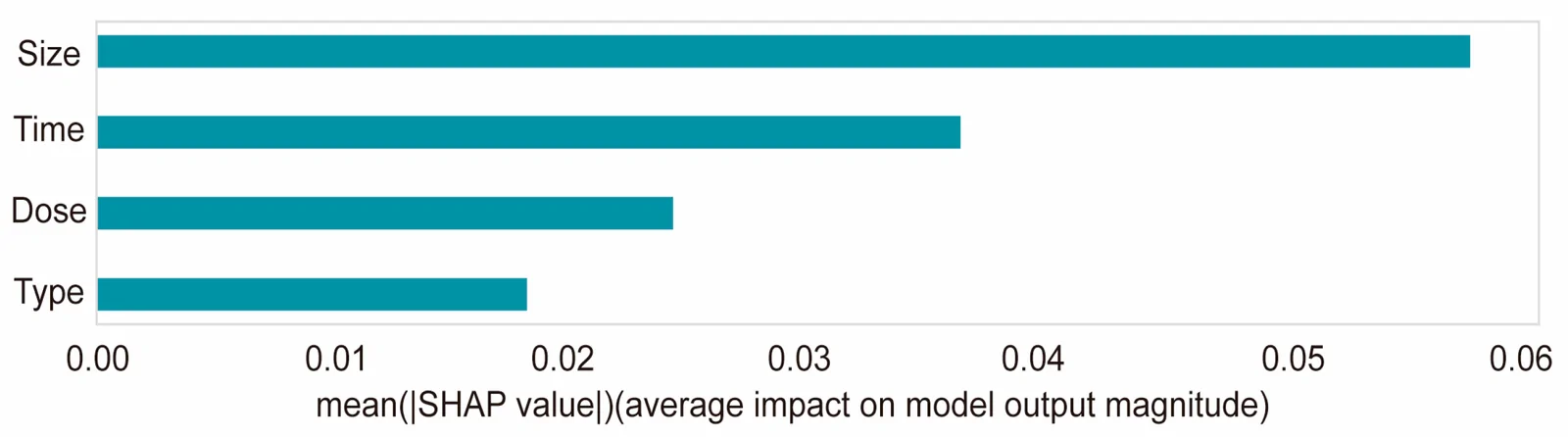
Fold-specific SHapley Additive exPlanations (SHAP) feature importance based on the XGBoost regression model. SHAP values were computed separately for each of the 19 Leave-One-Group-Out Cross-Validation (LOGO-CV) folds, and the mean absolute SHAP value was averaged across all folds.

To further reconcile the global feature importance with individual experimental observations, we employed the SHAP analysis. The SHAP summary plot for the XGBoost regression model (Figure 6A) identified exposure time as the most influential factor driving the numerical magnitude of enzymatic fluctuations, followed by dosage and size. Fold-specific SHAP values within the 19-fold LOGO-CV framework showed that size had the highest mean absolute SHAP value (0.054), followed by time (0.028), dose (0.024), and type (0.019) (Figure 7 and Table S10), consistent with the MDI results. This shift in ranking suggests that the relative contribution of exposure time may be partly influenced by between-study heterogeneity in the full dataset, whereas size remains the most robust predictor under strict source-grouped validation. To illustrate this context-dependent synergy, we utilized SHAP force plots to decompose the model’s logic for individual samples (Figure 6B,C). The plots quantify how each factor shifts the predicted enzyme activity from the base value (mean of the dataset) to the final output value:

Case I: Synergistic Promotion (Figure 6B). In a representative PE-type MP sample, starting from a base value of 0.970, the positive contributions from dosage (SHAP = +0.012), size (SHAP = +0.065), and exposure time (SHAP = +0.190) overcame the negative contribution from the type (SHAP = −0.007), driving the prediction to f(x) = 1.230 and pushing the final response toward enzymatic promotion.

Case II: Antagonistic Inhibition (Figure 6C). Conversely, for another PE sample starting from a base value of 0.970, despite a positive contribution from the type (SHAP = +0.008), the suppressive forces driven by size (SHAP = −0.100), dosage (SHAP = −0.016), and exposure time (SHAP = −0.012) dominated the prediction, resulting in a net inhibitory effect of f(x) = 0.850.

These localized interpretations bridge the gap between abstract model outputs and biological reality, providing a robust computational explanation for the heterogeneous and often contradictory enzymatic responses reported in MP literature.

## 4. Discussion

### 4.1. Comparison with Previous Studies

Unlike conventional meta-analyses or linear regression approaches that assume a consistent directional effect across studies, the ML framework adopted does not require pre-specified functional forms. As demonstrated by the correlation analysis, traditional linear methods revealed near-zero associations between individual MP properties and enzymatic activity. In contrast, the PDP and SHAP analyses uncovered distinct non-linear and interactive patterns that would have been missed under a conventional linear modeling framework, highlighting the added value of interpretable ML for hypothesis generation and mechanism discovery in environmental data synthesis. This conceptual advantage is further supported by a quantitative comparison: on 7 studies (169 effect sizes) with complete arm-level statistics, XGBoost achieved a 16.7% lower pooled RMSE than four-variable meta-regression (Figure S6).

We included a comparison with the work of Withana et al. [16], which provided an important foundation by applying ML to literature-derived microplastic-soil data; our study extends their approach in three aspects: (1) a broader enzyme panel covering 16 enzymes involved in C, N, and P cycling, versus a single enzyme; (2) explicit separation of direction (promotion vs. inhibition) from magnitude, combining XGBoost regression with RF classification; and (3) a rigorous 19-fold LOGO-CV grouped by DOI, which avoids the information leakage risk of random splitting and provides more conservative but realistic performance estimates.

### 4.2. Particle Size: A Key Factor Associated with Enzymatic Responses

Among the four MP properties, particle size showed the strongest association with enzymatic shifts among the evaluated features. The PDP analysis further revealed a robust, non-monotonic “size-sensitive window” across all three algorithms (XGBoost, RF, and GBR). The strongest inhibitory effect was consistently identified within the 200–600 μm range, while smaller and larger sizes exhibited relatively weaker suppression. This size-dependent response is supported by previous experimental evidence. Ma et al. (2023) reported that 300 μm PE exerted a stronger inhibitory effect on soil phosphatase activity than 600 μm PE [17]. One possible explanation is that particle size may influence MP-soil interactions and the resulting changes in the local soil physicochemical environment. Previous research has shown that MPs can affect extracellular enzyme activities through changes in soil physicochemical properties and direct interactions with enzymes [18]. Thus, the non-monotonic pattern observed here may reflect the combined effects of particle-soil interactions and differences in the soil microenvironment. Notably, the 200–600 μm range identified here should be interpreted as a dataset-derived sensitive range rather than a universal particle-size threshold. From an environmental monitoring perspective, these findings suggest that particle size should be considered an important descriptor when characterizing MP pollution in soil ecosystems. MPs within the observed 200–600 μm range were associated with stronger inhibitory responses in the present dataset than smaller or larger particles. Consequently, relying solely on bulk MP concentration may overlook potentially important size-dependent differences in soil biochemical responses. Incorporating particle-size information into future ecological risk assessments may therefore improve the characterization of MP-related risks to soil biochemical functioning.

### 4.3. Temporal Dynamics: Non-Linear Patterns in Enzymatic Responses

The PDP analysis revealed a non-linear temporal pattern, with greater variability during the early exposure period (0–105 days) followed by a gradual upward trend toward 310 days. This temporal dependence is consistent with experimental evidence showing that the effects of MPs on soil enzymatic activities can change during incubation. For example, Zhao et al. (2021) evaluated multiple MP shapes and polymer types at 3, 11, and 31 days, and found that some inhibitory effects were evident at early stages, whereas stimulatory effects emerged for certain enzymes at later stages [19]. The early-stage fluctuations likely reflect the dynamic response of soil biochemical processes to changing environmental conditions, although the specific mechanisms underlying the temporal pattern identified here cannot be determined from the present dataset. The subsequent upward trend toward 310 days may indicate a change in the magnitude or direction of the enzymatic response with prolonged exposure. It is important to note that our dataset is not longitudinal; the temporal patterns observed here are derived from comparing studies with different exposure durations rather than from repeated measurements within the same experiment. Therefore, these temporal patterns should be interpreted as cross-study observations, and establishing whether such adaptation occurs would require further time-resolved investigation [20,21,22].

### 4.4. Dose–Response Relationships: Non-Linear Patterns and a Dataset-Derived Plateau

Our results reconcile the seemingly contradictory findings in the literature regarding MP dosage. The PDP analysis revealed a non-linear dose–response relationship, with the predicted enzyme response tending to plateau at higher MP dosages. At lower dosages (≤20 g/kg), enzyme activity exhibited greater variation, while a slight upward trend was observed at the lowest dosages (<5 g/kg). This low-dose increase may indicate a potential stimulatory response, consistent with previous experimental studies [5,23] showing that MP exposure can stimulate certain soil enzyme activities and that enzymatic responses can vary with MP dose. As the dosage increased to 100 g/kg, the inhibitory response became less sensitive, and the PDP curve flattened. This plateau may reflect a plateau-like pattern, potentially related to the physical limit of MP retention within the soil pore structure. However, this mechanism cannot be directly demonstrated from the present dataset; it remains a plausible hypothesis rather than an established mechanism. Therefore, the approximately 100 g/kg level should be regarded as a dataset-derived transition toward a plateau rather than a universal threshold for MP toxicity. These findings indicate that MP effects on soil enzyme activity may not follow a simple linear dose–response relationship, highlighting the importance of considering dose-dependent non-linear responses when assessing the ecological effects of MP contamination.

### 4.5. Polymer Identity: Potential Physical and Chemical Contributions

Overall, MP type contributed only 11.0% to the global MDI ranking, placing it below size, dose, and time. However, this ranking should be interpreted with caution. MDI used in our feature importance analysis is known to favor predictors with higher cardinality or more split points. Here, size, dose, and time are continuous variables, while type was encoded via integer labels; thus, the relatively low importance of type may be partly methodological. The influence of PE may be primarily associated with its hydrophobicity and its potential to alter the physical structure of the soil matrix. Within the 200–600 μm size range, PE fragments may affect the spatial distribution of enzymes and substrates, thereby limiting their interactions and reducing enzymatic efficiency [24,25]. In contrast, PVC may additionally involve chemical processes. Its degradation can release additives and associated compounds, which may modify local soil conditions (e.g., pH) and influence enzyme stability or microbial activity [23,26]. It is important to note that SHAP and MDI quantify feature contributions to model predictions and do not establish causal relationships. The proposed mechanisms are therefore interpretive hypotheses rather than causal conclusions. Taken together, our results suggest that polymer-driven enzymatic responses may involve both physical and chemical processes. These findings underscore the necessity of incorporating polymer-specific traits into environmental monitoring and risk management [27].

### 4.6. Limitations and Future Perspectives

In this study, several limitations remain to be addressed. First, the model’s explanatory power is modest, with an R^2^ of 0.293 (±0.042), indicating that approximately 30% of the variance in enzymatic activity is explained by the four MP properties. Second, the stringent LOGO-CV demonstrated that out-of-domain extrapolation across independent literature sources remains challenging. The RF classifier’s AUC decreased from 0.814 under random splitting to 0.530 under LOGO-CV, falling to only marginally above chance. This indicates that the classification model cannot reliably predict the direction of enzymatic responses for unseen studies. Both limitations may be partly attributable to the influence of between-study heterogeneity and the exclusion of key covariates that were inconsistently reported across studies, including MP physicochemical properties (e.g., density, additives, and Young’s modulus) and soil-specific parameters (e.g., pH, SOM, and texture) [28,29]. Third, the per-enzyme analysis revealed substantial variability in model performance across enzyme types, with RMSE ranging from 0.049 (laccase) to 0.308 (phenol oxidase) (Table S11). While several enzymes with sufficient sample sizes (e.g., urease, *n* = 160; catalase, *n* = 115) showed improved predictive performance compared to the pooled model, 11 of the 17 enzymes had fewer than 30 records, limiting the robustness of enzyme-specific modeling. Fourth, independent double extraction was not performed and prediction intervals for individual estimates were not provided. Finally, the key patterns identified in this study (e.g., the size-sensitive window) remain data-driven and have not been mechanistically validated. Future work should prioritize (1) incorporation of soil physicochemical covariates and polymer-specific properties through standardized reporting to improve model explanatory power and generalizability; (2) per-enzyme modeling with larger sample sizes to enable more robust enzyme-specific predictions; (3) independent double extraction with reported inter-reviewer agreement to further minimize potential digitization errors; (4) application of uncertainty quantification methods, such as quantile regression forests or conformal prediction, to provide per-prediction intervals; and (5) mechanistic validation of the observed non-linear patterns through controlled microcosm experiments.

Nevertheless, our findings provide several implications for environmental monitoring and risk assessment in agricultural soils. First, particle size emerges as a prominent feature associated with enzymatic responses, suggesting that size distribution should be incorporated into monitoring protocols. Second, the observed temporal dynamics indicate that exposure duration critically influences biological responses, highlighting the importance of long-term assessments. Third, the non-linear dose–response relationships and polymer-specific effects suggest that risk evaluation frameworks should move beyond simple concentration-based metrics and consider multiple interacting factors. Finally, the modular design of our ML framework allows it to be extended to other particulate materials, such as bio-based and modified starches, provided that material-specific properties are incorporated as additional features and sufficient training data are available. Collectively, these insights contribute to a more nuanced understanding of MP-induced soil ecological risks and may inform more effective management strategies.

## 5. Conclusions

This study applied an interpretable ML framework to characterize the heterogeneous relationships between MPs and soil enzymatic activities. The results revealed weak linear correlations but substantial non-linear patterns across MP size, exposure time, dosage, and type. Among the evaluated features, size showed the highest model-based contribution, with a dataset-derived size-sensitive range of approximately 200–600 μm associated with stronger predicted enzymatic inhibition. Exposure time and dosage also showed non-linear patterns, including an upward trend in predicted responses toward longer exposure periods and a plateau-like pattern at dosages above approximately 100 g/kg. Overall, the interpretable ML framework provides quantitative insights into the heterogeneous and context-dependent enzymatic responses to MPs and helps identify response patterns and potential factors for further experimental investigation. The findings provide a basis for generating testable hypotheses regarding MP-soil enzyme interactions, while validation using controlled and longitudinal experiments is needed to establish the underlying mechanisms and assess their ecological significance.

## Data Availability

The original contributions presented in this study are included in the article/Supplementary Material. The original data presented in this study are included in the article and Supplementary Materials. The source code and analysis scripts are publicly available at Zenodo (DOI: 10.5281/zenodo.22855905). Further inquiries can be directed to the corresponding author.

## Supplementary Materials

The following supporting information can be downloaded at: https://www.mdpi.com/article/10.3390/biology15201880/s1. Figure S1. Overall research framework. Figure S2. Study-grouped out-of-fold permutation importance of microplastic properties. Figure S3. Bootstrap partial dependence plot (PDP) illustrating the non-linear effect of microplastic size on soil enzyme activity. Figure S4. Partial dependence plots (PDPs) within the Leave-One-Group-Out Cross-Validation (LOGO-CV) framework. Figure S5. Accumulated Local Effects (ALE) plots. Figure S6. Common-sample prediction comparison between meta-regression and XGBoost. Table S1. Physical and chemical properties of five polymer types included in this study. Table S2. Datasets used in this study. Table S3. Coefficient of determination (R^2^) for the six regression models. Table S4. Detailed evaluation metrics of 19-fold Leave-One-Group-Out Cross-Validation (LOGO-CV) by literature source. Table S5. Study-grouped out-of-fold permutation importance of microplastic properties across 19-fold Leave-One-Group-Out Cross-Validation (LOGO-CV). Table S6. Partial dependence values for size across three regression models. Table S7. Partial dependence values for dose across three regression models. Table S8. Partial dependence values for exposure time across three regression models. Table S9. Pairwise comparisons of partial dependence plot (PDP) values between the 200–600 μm window and adjacent size ranges. Table S10. Fold-specific SHapley Additive exPlanations (SHAP) values of microplastic properties across 19-fold Leave-One-Group-Out Cross-Validation (LOGO-CV). Table S11. Per-enzyme performance of the XGBoost regression model for individual soil enzymes.
