# Supplementary material for: Unraveling the Context-Dependent Effects of Microplastics on Soil Enzymatic Activities via Interpretable Machine Learning
Source: Biology (Basel). 2026 Oct 18;15(20):1880. doi: 10.3390/biology15201880 (PMC13701847; doi:10.3390/biology15201880)
Supplement: Supplementary file 1 [file biology-15-01880-s001.zip › Supplementary Figures.pdf]

Unraveling the Context-Dependent Effects of Microplastics on Soil Enzymatic Activities via Interpretable Machine Learning

Zifei Liu †, Yitong Liu †, Tianpeng Wang, Juanjuan Li, Xue Chi and Hong Li *

School of Life and Health Sciences, Hainan Province Key Laboratory of One Health, Collaborative Innovation Center of Life and Health, Hainan University, Haikou 570228, China

* Correspondence: lihongbio@hainanu.edu.cn

† These authors contributed equally to this work.

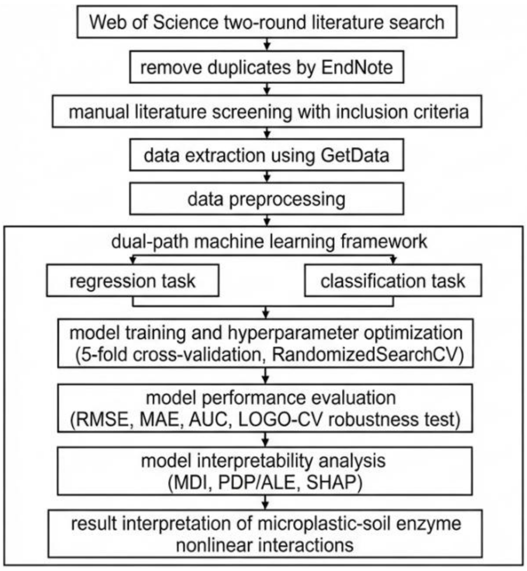

Figure S1. Overall research framework.

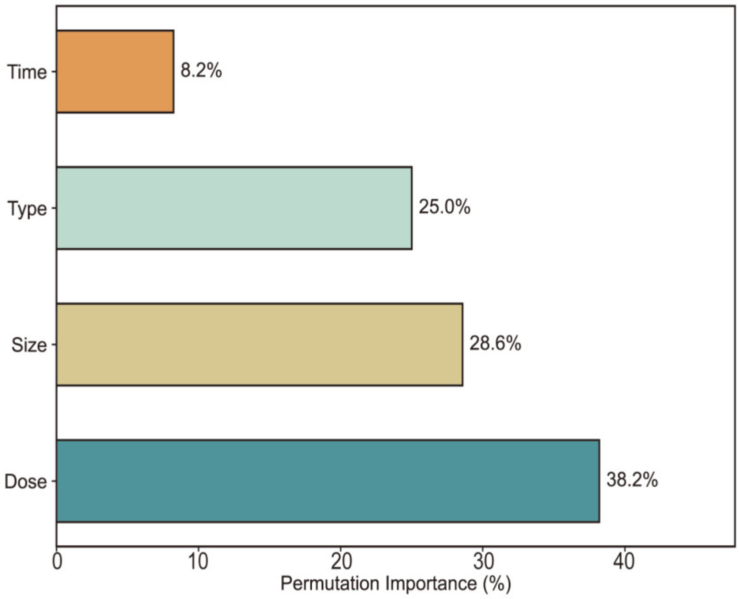

Figure S2. Study-grouped out-of-fold permutation importance of microplastic properties. Permutation importance was computed on the held-out fold within the 19-fold LOGO-CV grouped by DOI and averaged across all folds. Values represent the relative contribution (%) of each feature to the model’s predictive performance.

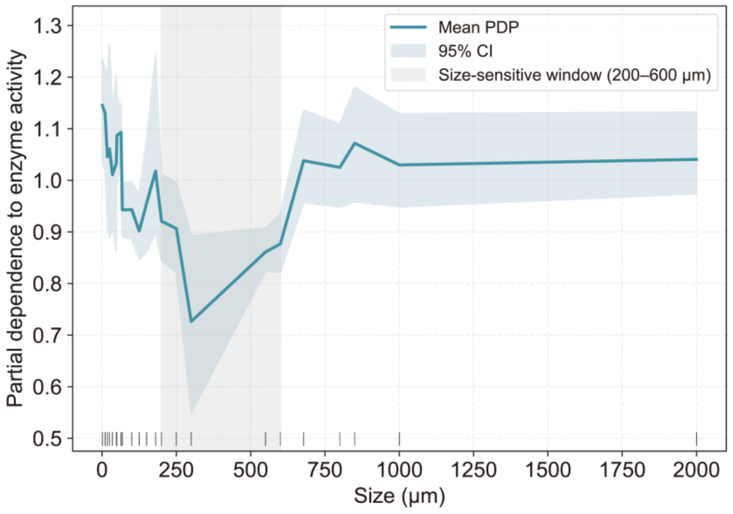

Figure S3. Bootstrap partial dependence plot (PDP) illustrating the non-linear effect of microplastic size on soil enzyme activity. Solid blue line represents the mean PDP derived from bootstrapping, and the light blue shaded area indicates the 95% confidence interval. The grey shaded region highlights the identified size-sensitive window. Vertical lines at the bottom indicate the actual data support (n = 592).

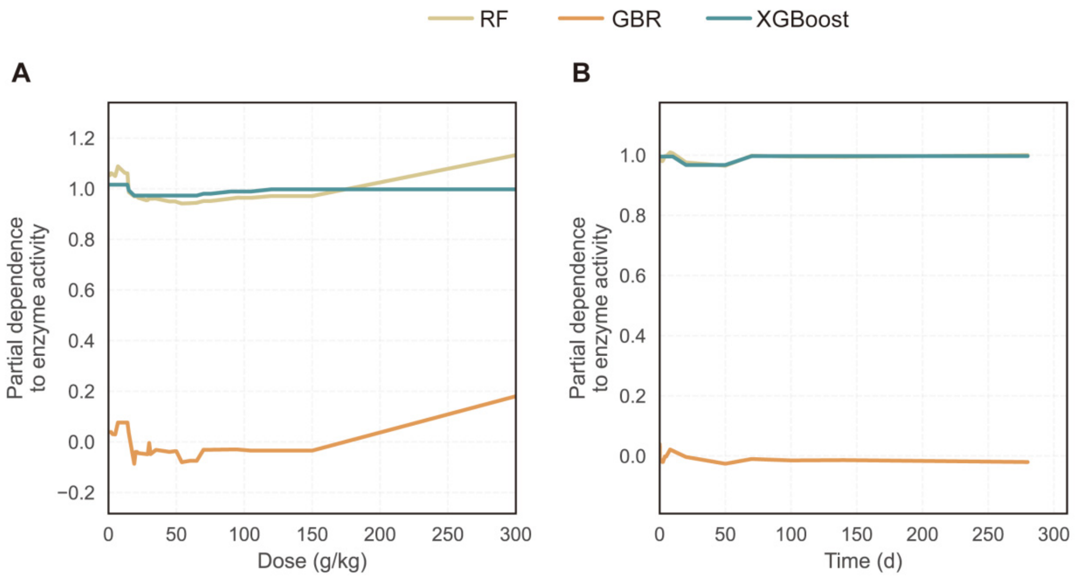

Figure S4. Partial dependence plots (PDPs) within the Leave-One-Group-Out Cross-Validation (LOGO-CV) framework.

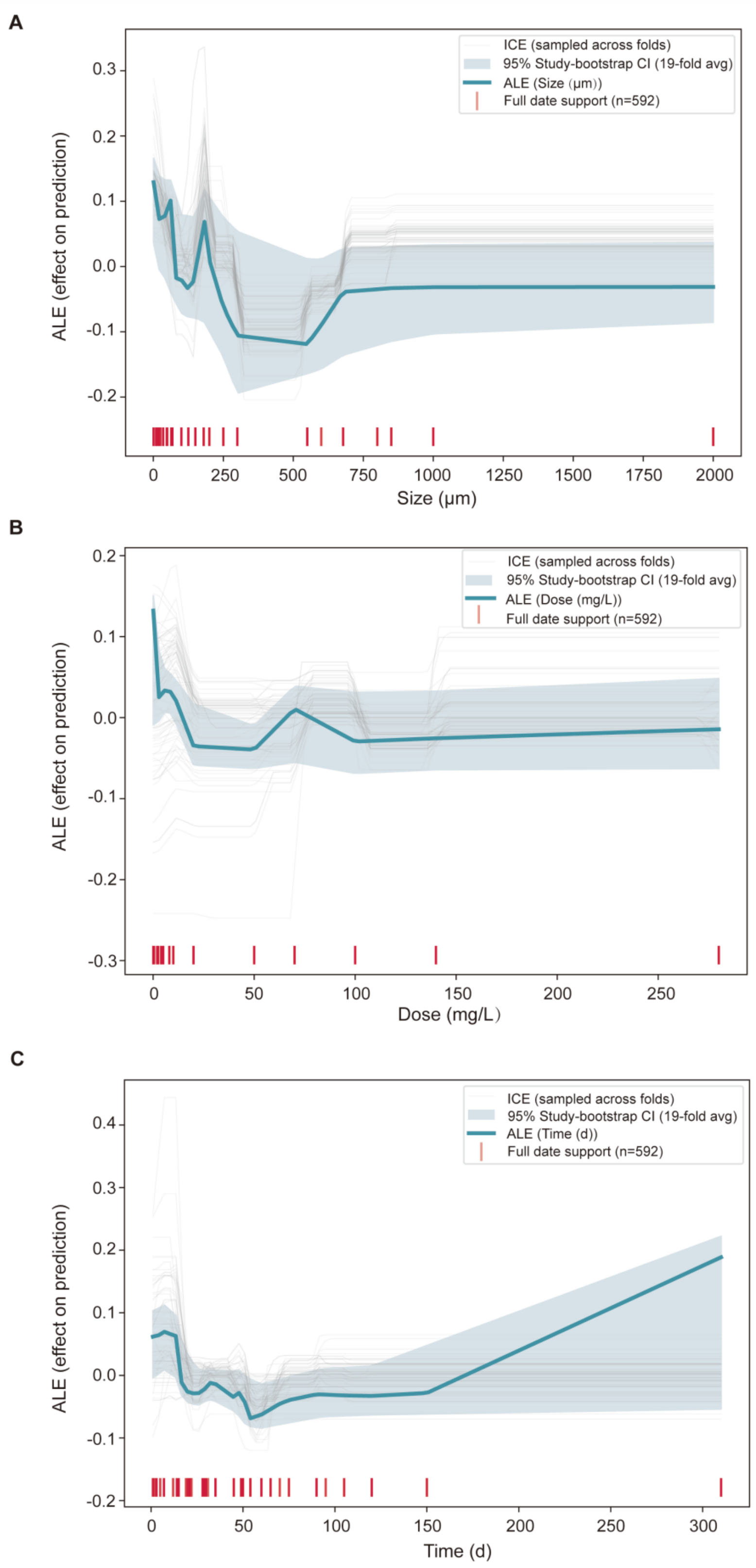

Figure S5. Accumulated Local Effects (ALE) plots for (A) size, (B) dose, and (C) exposure time. Solid lines represent the ALE curve; shaded bands represent 95% study-level bootstrap confidence intervals (19-fold average). Light gray lines

represent ICE curves sampled across the 19 LOGO-CV folds. Red vertical lines at the bottom indicate the actual data support (n = 592).

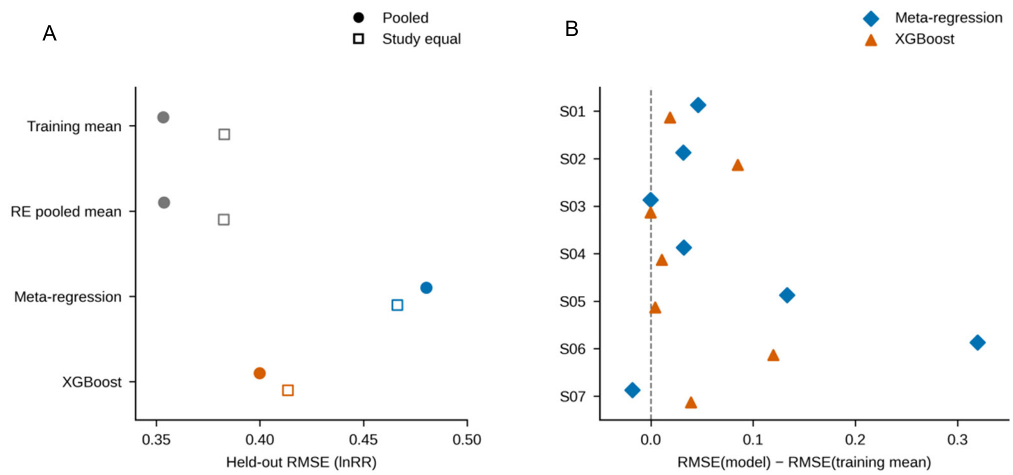

Figure S6. Common-sample prediction comparison between meta-regression and XGBoost on the 7 studies (169 effect sizes) with complete arm-level statistics. (A) Pooled and study-equal RMSE for the four models (training mean, random-effects pooled mean, four-variable meta-regression, and XGBoost). (B) Error difference between each model and the training-mean baseline for each held-out study (model RMSE − training-mean RMSE). Negative values indicate that the model outperformed the training-mean baseline.
